# Low-Temperature Stress during Capped Brood Stage Increases Pupal Mortality, Misorientation and Adult Mortality in Honey Bees

**DOI:** 10.1371/journal.pone.0154547

**Published:** 2016-05-05

**Authors:** Qing Wang, Xinjian Xu, Xiangjie Zhu, Lin Chen, Shujing Zhou, Zachary Y. Huang, Bingfeng Zhou

**Affiliations:** 1 College of Bee Science, Fujian Agriculture and Forestry University, Fuzhou, Fujian 350002, China; 2 College of Life Sciences, Fujian Agriculture and Forestry University, Fuzhou, Fujian 350002, China; 3 Department of Entomology, Michigan State University, East Lansing, MI 48824, United States of America; 4 Ecology, Evolutionary Biology and Behavior Program, Michigan State University, East Lansing, MI 48824, United States of America; 5 BEACON Center for the Study of Evolution in Action, Michigan State University, East Lansing, MI 48824, United States of America; University of North Carolina, Greensboro, UNITED STATES

## Abstract

Honey bees (*Apis mellifera*) are key pollinators, playing a vital role in ecosystem maintenance and stability of crop yields. Recently, reduced honey bee survival has attracted intensive attention. Among all other honey bee stresses, temperature is a fundamental ecological factor that has been shown to affect honey bee survival. Yet, the impact of low temperature stress during capped brood on brood mortality has not been systematically investigated. In addition, little was known about how low temperature exposure during capped brood affects subsequent adult longevity. In this study, capped worker broods at 12 different developmental stages were exposed to 20°C for 12, 24, 36, 48, 60, 72, 84 and 96 hours, followed by incubation at 35°C until emergence. We found that longer durations of low temperature during capped brood led to higher mortality, higher incidences of misorientation inside cells and shorter worker longevity. Capped brood as prepupae and near emergence were more sensitive to low-temperature exposure, while capped larvae and mid-pupal stages showed the highest resistance to low-temperature stress. Our results suggest that prepupae and pupae prior to eclosion are the most sensitive stages to low temperature stress, as they are to other stresses, presumably due to many physiological changes related to metamorphosis happening during these two stages. Understanding how low-temperature stress affects honey bee physiology and longevity can improve honey bee management strategies.

## Introduction

Honey bee (*Apis mellifera*) is one of the most important pollinators because of its major role in pollination [1], ecosystem stability and biodiversity [2–4]. However, honey bee survival is affected by many stressors. These include extreme temperatures [5, 6], poor nutrition [7, 8], pesticides [9, 10], parasites and pathogens [11, 12]. Temperature is the main ecological factor affecting the development of honey bee brood [13]. Nearly all of the non-social insects are poikilothermic, while many social insects can maintain precise nest temperatures. Honey bees have been known to regulate their nest temperatures with high precision [14, 15]. To ensure normal brood development, colonies spend much energy to maintain brood nest temperature in the range of 32–36°C [13, 16].

Many studies have shown that honey bee capped brood (larvae and pupal stages) raised at suboptimal temperatures can have negative effects on subsequent adult bees. First, brood raised at sub-optimal temperatures can result in deformations such as deformed wings, legs, and abdomen [17, 18]. Although eclosion rates are not different when brood are exposed to temperatures between 31–37°C, lengths of wings, proboscis, and tergum are significantly shorter and deformed bees appear at both ends of the extreme temperatures [6]. When pupae are exposed at 20°C for 96 h, eclosed Africanized honey bees (*A*. *mellifera scutellata*) show high percentages of split stings and deformed wings [19]. In Western bees the low temperature results in imperfection in wing veins [20, 21] and larger angle G18 in forewings [22]. Numbers of microglomeruli in the olfactory lobes are highest in bees raised at 34.5°C as pupae and are reduced in bees raised below or above this norm [5]. Secondly, adult bees raised at lower temperatures also show differences in behavior, learning and memory. Bees raised at 35°C and 36°C as brood show a better learning and memory than those raised at 32°C and 33°C [23]. Bees raised at 32°C as pupae are poor dancers and learners, and they disappear soon after orientation flight [24]. Low brood temperature also causes reduced probability of workers performing dancing and undertaking, and a delayed age for foraging [25]. Finally, bees raised at lower brood temperature are also more susceptible to stresses. For example, bees emerging from 33°C are more susceptible to pesticides [26]. Similarly, tracheal mite prevalence level is doubled in adult bees when pupae are raised at 30°C for 9 days [27].

Despite of the large number of studies concentrating on effects of brood temperature on morphology and behavior, no studies have systematically explored the effects of low temperatures on mortality of brood and survival of eclosed adults. Our study attempted to answer two questions: 1) which capped brood stage is more sensitive to cold stress? And 2) how does low temperature during capped brood affect longevity of eclosed adults. Our aim is to understand the effects of low-temperature stress on honey bee survival so that these temperature-duration combinations can be avoided during beekeeping.

## Materials and Methods

### Honey bee worker capped brood

Experiments were conducted in the summer of 2014 in Fuzhou, China (GPS coordinates: (N 26°05’, E 119°13’). Colonies of Western honey bees (*Apis mellifera*) were healthy and each colony had approximately 20,000–30,000 workers. To obtain brood of specific ages, queens were individually confined to an empty comb inside a cage. These cages had queen-excluding grids, which confined the queens within but allowed workers to move freely in and out of the cages. Eight days later, we identified newly capped brood cells by mapping twice the brood cells that were ready to be capped (at time 0 and then 4 h later). For each treatment, a patch of comb containing 7–38 newly capped brood cells (within 4 h) was cut out and brought into the lab immediately (starting time of L0). The experiment was repeated using three different colonies. Each piece of brood was stationed in the normal orientation inside the incubators. All the control groups were kept at 35°C until emergence.

### Low temperature treatment

**Fig 1:**
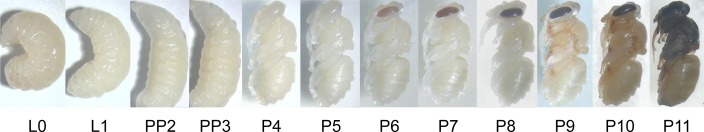
Morphology and color changes during the 12 days of capped brood. Larval (L) stage has 2 days (because we included the 0 h capped brood as the same day of being capped, L0), prepupae (PP) has 2 days and the rest are pupae (P4-P11). On day 12 the worker would eclose (emerge) as an adult.

Different developmental stages of capped brood were treated at 20°C (CTHI-250B, Stik Group LLC, USA; precision, ±0.1°C) for different durations. Except during cold stress, brood cells were kept at 35°C. We designated the 12 different days of capped brood as L0-L1 (day 0 and 1 during larvae), PP2-PP3 (2 days of prepupae) and P4-P11 (8 days as pupae) (Fig 1). The letter(s) represent developmental stages while numbers represent days after capping. L0 stood for larvae that was within 24 h of being capped (the capping happened during a 4 h window). P11 was the day before emergence. The brood cells were exposed to 20°C for either 12, 24, 36, 48, 60, 72, 84, or 96 h.

### Temperature monitoring

During the entire incubation period, data loggers (HC-02, Hangzhou Hongchang Technology, China) recorded temperature and relative humidity (RH) for each incubator at 10 min intervals. The actual temperature and RH of 35°C and 20°C incubators were 34.93±0.00°C, RH 75.48±0.01% and 19.97±0.00°C, RH 74.37±0.01% (mean ± SE), respectively. Because we had 12 stages and 8 different durations and 3 replicates (colonies), we had a total of 5,953 honey bees for mortality and survival analysis.

### Brood mortality and adult longevity

To calculate mortality, we counted both the number of cells with dead brood and newly emerged bees. Capped cells were considered dead if they did not emerge 17 days post capping (about 5 days later than normal eclosion) unless they eclosed but with head in the wrong direction (see below). After emergence, bees were marked with color (SP-110, Sino Path Enterprises LTD, China) according to colony source, caged inside wooden cages (dimensions: 12.0 x 10.0 x 12.0 cm) and brought into humidity- and temperature-controlled incubators (dark, 33°C and 60% RH) as described by [25]. Thus bees from each treatment from all three colonies were caged together but bee colors allowed us to know the colony source of workers. On average 45 adult bees per treatment were evaluated for longevity, but due to high brood mortality, some treatments had lower adult bee numbers. We did not use those treatments which had less than 15 adult bees combined from all three colonies in survival analysis. All of the caged bees were fed with 50% sucrose solution and water in vials, pollen mixed with 50% sucrose solution in tube. The food was replaced every three days. We recorded the number of dead bees and removed them daily.

### Misorientation

When capped brood were checked for mortality, many bees from cold-exposed L1 (24 h after capping) were found to orient their heads the wrong way, causing the bees to die in the cells because the emerged workers could not chew their way out. To determine the brood stage that were most sensitive to cold to result in misorientation, we used capped brood (N = 86–103 pupae per treatment) that were 4, 8, 12, 16, 20, 24, 28, 32, 36 and 40 h after capping and exposed them at 20°C for 2 h. After determining the most sensitive stage, we then exposed the stage at 20°C for either a short exposure (5, 10, 30, 60 and 90 min) (N = 93–112 pupae per treatment) or a long exposure (12, 24, 36, 48, 60, 72, 84 and 96 h) (N = 49–80 pupae per treatment) to see which duration results in the highest misorientation. We determined the misorientation rate if they did not emerge 17 days post capping by uncapping each cell and recording their orientation.

### Statistical analyses

Mortality data were analyzed as a split-plot with developmental stages as main plots and cold durations as subplots [28], after transformation of arcsine (square root) of the mortality data. We determined the 50% lethal time (LT_50_) for each stage by using PoloPlus (LeOra Software Company), a software normally used for LD_50_ determination using Probit analysis. One-Way ANOVA was used after transformation of arcsine (square root) for misorientation data. When ANOVA detected a significant effect, we used Student–Newman–Keuls tests to conduct pair-wise comparisons among treatments. Kaplan Meier survival analyses (Log rank, with options for “survival tables, mean and median survival”) were used to estimate effects on lifespan of all groups [29]. We tested for differences in survival among groups by Log-rank tests. ANOVA and survival analyses were done using SPSS 13.0.

## Results

### Mortality and LT_50_ of different brood stages

**Fig 2:**
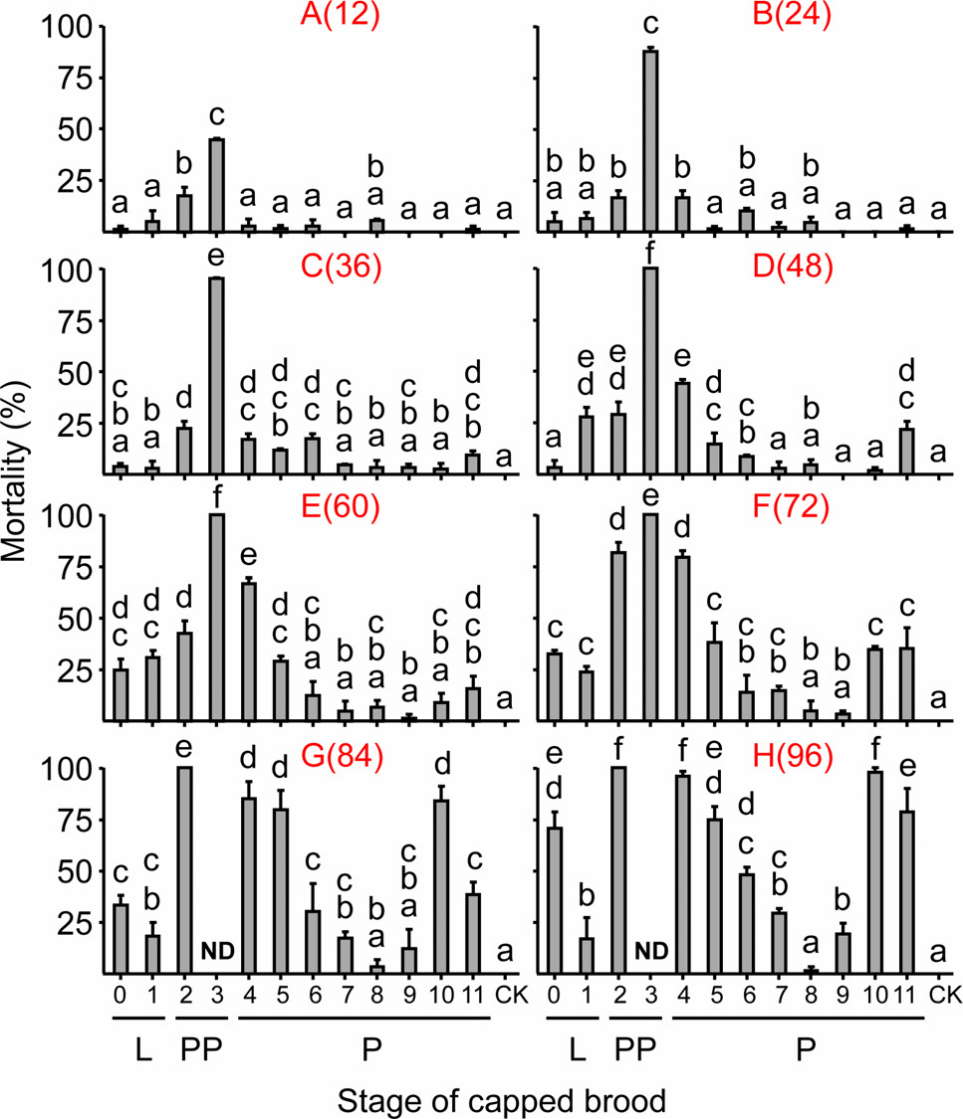
Mean mortality (±SE) of capped brood. Each stage was treated with 20°C for different durations (with number of h indicated in parenthesis). Please refer to Fig 1 for age of different groups. Means were based on 3 colonies. Different letters on top of each bar indicate that they are statistically different from others by Student–Newman–Keuls tests after ANOVA showed a significant difference. Control group was indicated as “CK”. ND: not determined because this treatment at shorter durations (48–72 h) all died.

Capped brood of different stages and different cold-exposure duration showed a significant interaction (F = 6.82; df = 64, 128; P = 0.000) (Fig 2).

Late prepupae (PP3) had the highest mortality in all low-temperature treatments (Fig 2), reaching 100% mortality in exposure of 48 h or longer. PP2 also showed a similarly high mortality at exposure of 72 h or longer, reaching 100% at the two longest exposures (84 and 96 h). This is followed by P4 and P5 at durations of 72 h or longer (Fig 2F–2H), but at shorter durations (<60 h) they were quite resistant to cold (Fig 2A–2D). P10 and P11 had high mortality when cold duration was longer than 72 h. L0 only had high mortality at the longest cold duration (Fig 2H) and showed similar mortality as control (CK) if cold duration was 48 h or shorter. L1 showed higher mortality than control when cold duration was at 48 h or longer. P8 had the lowest mortality across all cold durations, their mortality was not significantly different from control (P > 0.05) at all durations. P9 showed an increased mortality only at 96 h. LT_50_ showed a similar pattern. For example PP3 had the shortest LT_50_ (19.3 h) (Table 1). PP2 and P4 also showed low LT_50_ values, with 48.3 h and 47.6 h, respectively. P6-P9 all had LT_50_ longer than 96 h followed by L0 (87.2 h), P11 (79.3 h) and P10 (73.8 h).

**Table 1:** Time (h ± SE) required to kill 50% of pupae at 20°C (LT_50_) for different stages of sealed brood.

Stage	N	LT_50_ (h)	SE
L0	385	87.2 c	5.6
L1	441	> 96 d	
PP2	431	48.3 b	4.2
PP3	294	19.3 a	0.9
P4	461	47.6 b	2.7
P5	427	70.8 c	0.8
P6	464	> 96 d	
P7	466	> 96 d	
P8	439	> 96 d	
P9	428	> 96 d	
P10	422	73.8 c	1.0
P11	408	79.3 c	8.1

LT_50_ followed by different letters indicates a statistical difference (*P* < 0.05) by Student–Newman–Keuls tests after ANOVA showed a significant difference.

### Longevity of adult bees

**Fig 3:**
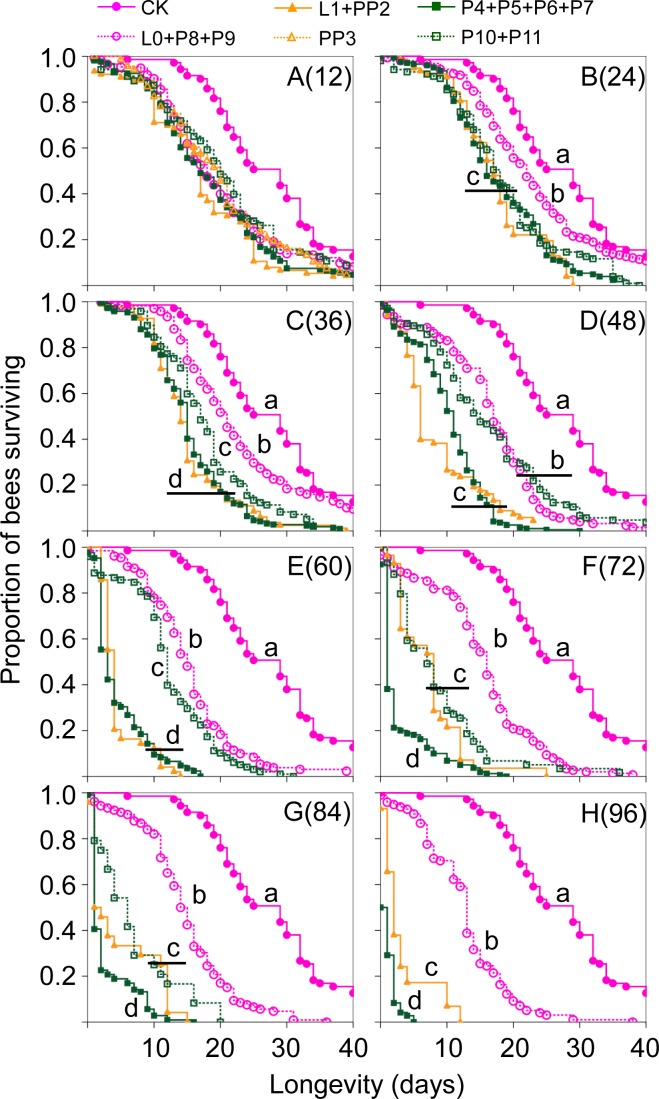
Kaplan Meier survival curves of adult bees after they were treated with cold exposure during brood stages. Different durations of 20°C were presented in A to H, with number of h indicated in parenthesis. Not enough PP3 survived to adults for survival analysis except in A(12). We pooled the 11 different stages into 4 groups because these 4 groups did not show differences in their survival. Please refer to Fig 1 for age of different groups. Each curve based on workers from 3 different colonies. Control group was indicated as “CK”. Kaplan Meier survival analyses were done on data 70 days post emergence but only 40 days of data are shown here for clarity. For A(12), separation of curves were: CK a, P10+P11 b, L0+P8+P9 bc, PP3 bcd, P4+P5+P6+P7 cd; L1+PP2 d. Not enough adults emerged (<15 for B(24), and 0 for others) at 24 h or longer for PP3, so no data for PP3 is presented except for A(12).

The mean lifespan of adult bees exposed to cold treatment was significantly shorter than that of the control group (Table 2). Log-rank tests showed no significant differences among L0, P8, and P9, between L1 and PP2, among P4, P5, P6 and P7, and between P10 and P11, in all the different durations. Therefore, the data within these four groups were pooled for analysis (Fig 3).

**Table 2:** Mean longevity in days (±SE) of various aged bees (vertical) exposed at 20°C for different periods (horizontal).

Stage	Exposure duration (h)							
	12	24	36	48	60	72	84	96
L0+P8+P9	21.0±0.9	23.9±0.9	23.5±0.9	17.0±0.7	15.7±0.7	15.6±0.6	14.9±0.7	12.9±0.6
L1+PP2	17.5±1.1	17.4±0.7	15.2±0.7	9.5±1.1	4.6±0.4	7.3±1.0	4.9±1.1	3.4±0.7
PP3	20.5±1.2							
P4+P5+P6+P7	18.8±0.7	18.4±0.6	15.4±0.4	10.4±0.4	4.8±0.3	2.9±0.3	2.8±0.3	0.9±0.1
P10+P11	21.2±1.1	18.6±0.9	18.4±0.9	17.0±1.1	12.5±0.7	8.9±1.0	7.0±1.2	
CK	28.4±1.3							

Data based on estimates from Kaplan Meier survival analysis by SPSS. Statistical differences among the groups are indicated in Fig 3.

After the shortest exposure of 12 h, all treatment groups differed from the control in survival (Fig 3A). P4+P5+P6+P7 had the shortest survival time when cold treatment was at 72 or longer (Fig 3F–3H), but it was indistinguishable from L1+PP2 in shorter exposures (Fig 3A–3E).

Bees from early capped larva and mid-pupa (L0+P8+P9 group) showed the best survival compared to others in nearly all durations (except Fig 3A). These bees had the most similar lifespan compared to control bees but they were still significantly shortened compared to the control at all durations (Fig 3A–3H), at 96 h the shortening of longevity was more than 15 days compared to the control (Fig 3H).

### Misorientation

**Fig 4:**
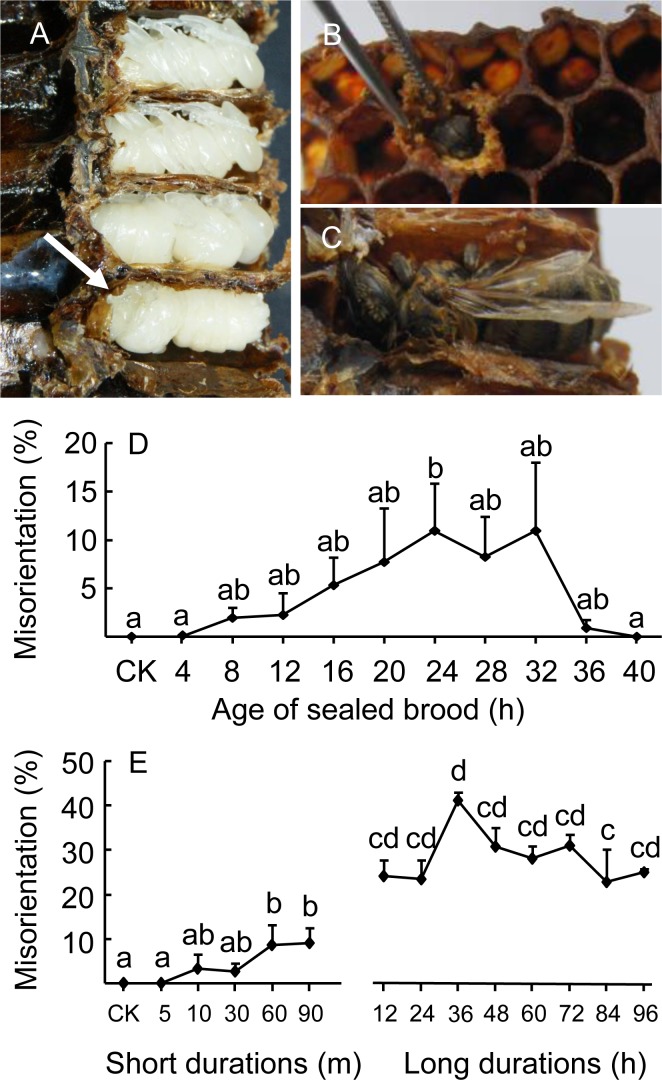
Misorienting pupa and rates of misorientation after cold treatments. (A) Misorientation of a pupa, (B) ofan eclosed bee and (C) the same misorientated bee shown sideways). (D) Rate of misorientation of different aged brood (±SE) after they were exposed to 20°C for 2 h. (E) Rate of misorientation after 24 h old brood (L1) were exposed to different durations of 20°C. These misoriented bees died due to starvation, even though technically they survived until eclosion. Control group was indicated as “CK”.

Exposure to cold of capped brood caused misorientation, which resulted in normal eclosion but the emerged bees could not exit their cells because their heads were toward the cell bottom (Fig 4A–4C). Brood at 24 h were the most sensitive to a 2 h cold exposure, with to a maximum of about 10% of misorientation (Fig 4D). We therefore used 24 h brood to be exposed at different cold durations. Cold exposure at durations of 60 or 90 m caused significantly higher rates of misorientation (~10%) compared to the control (“CK”), which had 0% misorientation. Longer durations of 24 h brood to cold caused much higher rates of misorientation, with 36 h exposure reaching a rate of 41.1% misorientation (Fig 4E). All treatments of the “long durations” caused significantly higher rates of misorientation compared to the highest rate of “short durations” (10% at 90 m).

## Discussion

The major findings of this study are that 1) For brood mortality, the most sensitive period to cold temperature stress was PP3, followed by PP2 (Fig 2). Other stages were duration dependent: at exposures longer than 72 h, P10 and P11 were the most sensitive, but at durations shorter than 72 h, P4 was more sensitive. 2). The most resistant stage during all exposure durations was P8, followed by P9 and L0. 3). Adult bees emerged from cold-treated pupae showed reduced longevity. Adults emerged from L0 + P8 + P9 had the longest survival, but still significantly shorter compared to the control (CK, pupae not exposed to cold). 4) Cold temperature exposure to capped brood caused pupae to misorient such that they could eclose normally but still died inside cells, due to starvation (Fig 4A–4C). Brood 24 h post capping were the most sensitive and the rates of misorientation reached about 40% at this stage, if exposure was for 36 h.

Prepupa is the stage just before pupation and many internal organs are reorganized during this period [30]. Therefore honey bee prepupae are known to be sensitive to both temperature changes and to vibration [31, 32]. Our results are consistent with these observations because PP3 and PP2 were the most sensitive period, not only showing higher pupal mortality (Fig 2), but also shorter lifespan when adults emerged from cold treated pupae (Fig 3). The next most sensitive periods are P10 and P11, 1–2 days before eclosion and P4, one day after pupation (Fig 2). Thus stages associated with metamorphosis, such as prepupae (PP2 and PP3), 1 day after pupation (P4), or 1–2 days before eclosion (P10 and P11), are more sensitive to cold stress. Adult survivals are similar to this pattern, with PP3 being most sensitive and L0 and P8, P9 the most tolerant (these groups did not separate statically, Fig 3). PP3 showed the highest sensitivity because after 24 h of exposure, PP3 had fewer than 15 bees emerged, and no bees emerged after longer exposure; so the survival curve data was mostly missing (except at 12 h) because of their higher sensitivity. Only one exception is P4+P5+P6+P7 together, they appeared to be more sensitive than P10+P11 (Fig 3), while during brood P10+P11 had much higher mortality, especially during 84 and 96 h exposures.

Western honey bees evolved in the tropics and have adopted the strategy to maintain their brood at a high temperature of 35°C. So it is not surprising that brood kept at lower temperatures experience mortality. Weiss [33] showed that larvae also experience high mortality at low temperatures (5–24°C), especially after 72 h exposure. Our data show that under 12 h of 20°C cold exposure, bees experienced very little mortality during pupal stage if they were 4 days post capping (P4), i.e. right after pupation. Even at 24 h or 36 h exposure, the mortality of P4 to P11 were all below 20% and perhaps sustainable from the colony’s point of view. Despite all the honey bee studies related to cold exposure [5, 6, 19–26], we know little about the mechanisms at the physiological level. Chilling injury in other insects is shown to negatively affect ion channel function, preventing normal functions of muscle and nervous system [34]. A recent study in *Megachile rotundata* showed low temperature stress (6°C) to pupae led to decreased metabolic rates and flight performance in males, and reduced feeding and activity levels in both sexes; again suggesting neurological damages [35]. In adult *Drosophila*, multiple cold shocks caused differential regulation in several genes related to muscle function [36]. It is possible that low temperature stress during pupae affects adult muscle development, because even for adult honey bees, high temperature (35°C) is required for flight muscle maturation (Z.Y. Huang, unpublished data). In addition many enzymes involved in critical functions of honey bees, such as enzymes for juvenile hormone and ecdysteroid synthesis, might be impaired at low temperatures, leading to pupal death and also later effects on adults.

However, although most of these bees at P4-P11 survived to eclosion and had normal morphology after cold treatment, their lifespan was still significantly reduced. For example, pupae with 12 h cold exposure showed low mortality (Fig 2A) but had significant adult longevity reduction compared to the control (Fig 3A). Again we do not understand the underlying mechanisms why adult bees raised at lower temperatures will appear normal but live shorter lives. However our data are consistent with the observations that bees developed under 32°C disappeared soon in the hive [24]. Longevity of adult bees is a key factor affecting colony health. Short-lived workers can have a significant impact on colony health, resulting in shortage of foragers, thus disrupting the age-based division of labor. In this case, nurse bees will be forced to become foragers early, the impact on colony by earlier foraging are equivalent to a 5 fold increase of forager mortality according to a simulation [37]. Disrupting the age-based colony structure can potentially result in severe long-term consequences for food processing, brood care, foraging efficiency and other parameters [38–39]. This will obviously cause reduced honey production, although there are yet no studies to link the two. Furthermore, shorter worker longevity will cause earlier foraging in younger bees and this acceleration causes a positive feedback loop to result in colony failure [40].

It is not clear why honey bee larvae show a much higher rate of misorientation when brood were exposed to low temperature. Honey bee larvae have been shown to be sensitive to texture during their turns (“somersaults”) when spinning their cocoon, to make the correct orientation so that they can subsequently chew their way out [41]. If they become misoriented during pupation, there is not enough room in a cell for the adult to turn around, so they die from starvation. It is possible that under lowered temperature, bee larvae reduce their ability to detect the texture differences of the two cell ends. Alternatively, they might reduce their number of turns due to cold temperature so some of them end up at the wrong orientation only because they could not make another turn. From a colony’s point of view, these bees are not different from non-eclosed, dead bees, because they cannot successfully emerge from their cells. Indeed, if we include the misoriented bees at L1, then the L1 true mortality will be much higher. For example at shorter durations (< 72 h or shorter), L1 had higher true mortality than PP2.

It appears that during the evolutionary process honey bee brood are “spoiled” by eusocial thermoregulation behavior. Because honey bee brood are maintained at 33°C-35°C, outside that range they suffer a fitness loss [23]. It seems that honey bee brood and young bees have been adapted to the constant warm temperature and become stenothermic. For example, newly emerged bees cannot fly and need about 7 days to develop their flight muscles. This muscle development requires 35°C, because newly emerged bees, if maintained at 25°C for 7 day old bees, were not be able to fly (Z. Y. Huang, unpublished data). Therefore honey bee brood may lose the abilities to cope with abnormal temperatures beyond its nest norm. In contrast, solitary insects have much stronger abilities to cope with harsh temperature stress. For example, solitary leafcutter bees *Megachile rotundata*, a low temperature of 6°C has to be used during pupal stage to cause observed effects in changes in adult morphology and behaviors [35]. It will be interesting to determine if the opening nesting honey bees (*Apis dorsata* and *A*. *florea*, and their sister species) show higher tolerance to low temperature stress, compared to *Apis mellifera*. Alternatively, bumble bees could be used in a comparative approach. Their nest temperature is not regulated as precisely so they should be more cold-tolerant compared to honey bees.

It is highly unlikely that temperature would drop to 20°C for such a long time (12 h or longer) inside honey bee colonies, unless most of the workers are lost in a short time (e.g. due to a pesticide kill). Our study was mainly designed to determine how these extreme conditions may affect honey bee mortality and development. Understanding the consequences of these events should help us better manage the honey bees during beekeeping and let us know what low temperature and duration to avoid when we manage colonies under low temperatures during beekeeping.

## Supporting Information

S1 Table Dataset used for analysis and Figs 2–4. (XLS)
